# Effect of electroacupuncture on postoperative delirium in elderly patients undergoing laparoscopic radical prostatectomy: study protocol for a double-center randomized controlled trial

**DOI:** 10.3389/fmed.2026.1790084

**Published:** 2026-04-01

**Authors:** Shirong Wei, Sitong Zhou, Tong Zhi, Yungong Wang, Chaobo Ni, Zhangtian Xia, Tesheng Gao, Ming Yao, Huadong Ni

**Affiliations:** 1 Department of Anesthesiology and Pain Research Center, The Affiliated Hospital of Jiaxing University, The First Hospital of Jiaxing, Jiaxing, China; 2 Department of Anesthesiology, Jiaxing Hospital of Traditional Chinese Medicine, Jiaxing, China

**Keywords:** electroacupuncture, frail elderly, postoperative delirium, prostate cancer, study protocol

## Abstract

**Background:**

Postoperative delirium (POD) is an acute disorder of attention and cognition, frequently observed as a complication in elderly individuals undergoing major surgery. Patients undergoing laparoscopic radical prostatectomy (LRP) are particularly vulnerable due to advanced age and comorbidities. Electroacupuncture (EA), as a non-pharmacological intervention, may attenuate neuroinflammation and cerebral injury, potentially reducing the risk of POD. This study aims to evaluate the efficacy of EA in reducing the incidence of POD among elderly patients undergoing LRP.

**Methods:**

This is a double-center, prospective, randomized, patient- and assessor-blinded, sham-controlled trial. A total of 212 eligible participants will be enrolled and allocated in a 1:1 ratio to either the EA group or the sham EA (SEA) group. Participants in the EA group will receive intervention at the acupoints Zusanli (ST36) and Baihui (GV20), initiated 30 min prior to anesthesia induction and maintained for 25–30 min. The primary outcome is the incidence of POD assessed using the 3D-CAM during the first three postoperative days. Secondary endpoints include the duration and severity of delirium, delirium subtypes, pain scores, opioid consumption, catheter-related bladder discomfort, adverse events, and plasma levels of neuroinflammatory biomarkers.

**Discussion:**

Currently, evidence regarding the efficacy of EA in preventing POD specifically in elderly patients undergoing LRP is limited. This randomized controlled trial is designed to address this research gap. If the hypothesis is confirmed, this study will provide evidence for a safe, non-pharmacological strategy to improve postoperative cognitive outcomes in this population.

**Clinical trial registration:**

https://www.chictr.org.cn/showproj.html?proj=240562, Identifier: ChiCTR2500097337.

## Introduction

Delirium is characterized by an acute confusional state marked by abrupt onset of confusion, fluctuating course, inattention, and often altered consciousness (1). POD is a common and serious surgical complication among older patients after surgery (2). A growing body of evidence indicates the strong association between delirium and adverse patient outcomes, including prolonged hospitalization, increased complications, long-term cognitive dysfunction post-surgery, and heightened mortality risk (3, 4). These findings suggest that the prevention and management of POD warrant clinical attention.

Prostate cancer is the second most common cancer and the fifth-leading cause of death from cancer among men (5). Most prostate cancer patients are elderly, who often have more comorbidities and a higher incidence of POD. Previous studies have reported that the incidence of POD in patients undergoing radical prostatectomy ranges from 15.4% to 39.3% (6–8). The United States Cancer Statistics for 2020 revealed that the median age stood at 66 years for prostate cancer (9, 10). As the gold standard for treatment of prostate cancer, in LRP, patients are typically positioned in the Trendelenburg position and require sustained pneumoperitoneum, both of which can increase the risk of POD. Meanwhile, the complexity of cancer radical surgery and the potential postoperative pain may still trigger an inflammatory response, thereby impacting cognitive function (11, 12).

Currently, it is believed that multi-component non-pharmacological interventions (such as the ABCDEF bundle care, pain management, early mobilization, and adequate fluid supplementation) are the most effective methods for reducing POD (13–15). Regarding pharmacological interventions, recent meta-analyses indicate that α_2_-adrenoceptor agonists (specifically dexmedetomidine) are among the most effective strategies for preventing POD. In contrast, evidence supporting the prophylactic use of other agents, such as benzodiazepines, remains insufficient (16, 17). EA, a treatment method that combines traditional acupuncture with modern electrical stimulation techniques, has shown potential in the treatment of cognitive dysfunction in recent years (18, 19). A recent umbrella review also noted that acupuncture was associated with a reduction in postoperative neurocognitive complication (20). However, the mechanism of EA in preventing POD is not yet clear. According to recent studies, EA can inhibit the expression levels of pro-inflammatory cytokines, including IL-1*α*, IL-6, and TNF-α, to counteract neuroinflammation and oxidative stress and alleviate cognitive impairment (21). Phosphorylated tau protein 217 (P-tau 217), a key biomarker closely associated with the pathological mechanisms of neurodegenerative diseases such as Alzheimer’s disease (AD), may also be related to POD. Yet, the correlation between plasma P-tau 217 levels and POD following EA treatment has not been verified and warrants further investigation (22).

As a non-pharmacological intervention, the effectiveness of EA in reducing the incidence of POD has been demonstrated in previous studies, though most of the research has focused on cardiac and lower limb joint surgeries (23, 24). Currently, studies targeting elderly patients with prostate cancer remain limited. Therefore, there is an urgent need for randomized controlled trial to evaluate the efficacy of EA in treating POD following LRP. We hypothesize that EA may mitigate POD in this cohort. Specifically, the acupoints Zusanli (ST36) and Baihui (GV20) were selected for this protocol based on their purported roles in regulating inflammatory responses and improving cerebral function (25), respectively. To test this hypothesis, we designed a double-center, randomized, sham-controlled trial. This study aims to evaluate the efficacy of EA in preventing POD in elderly patients undergoing LRP, employing a methodological framework to minimize bias.

## Methods and analysis

### Trial setting

**Figure 1:**
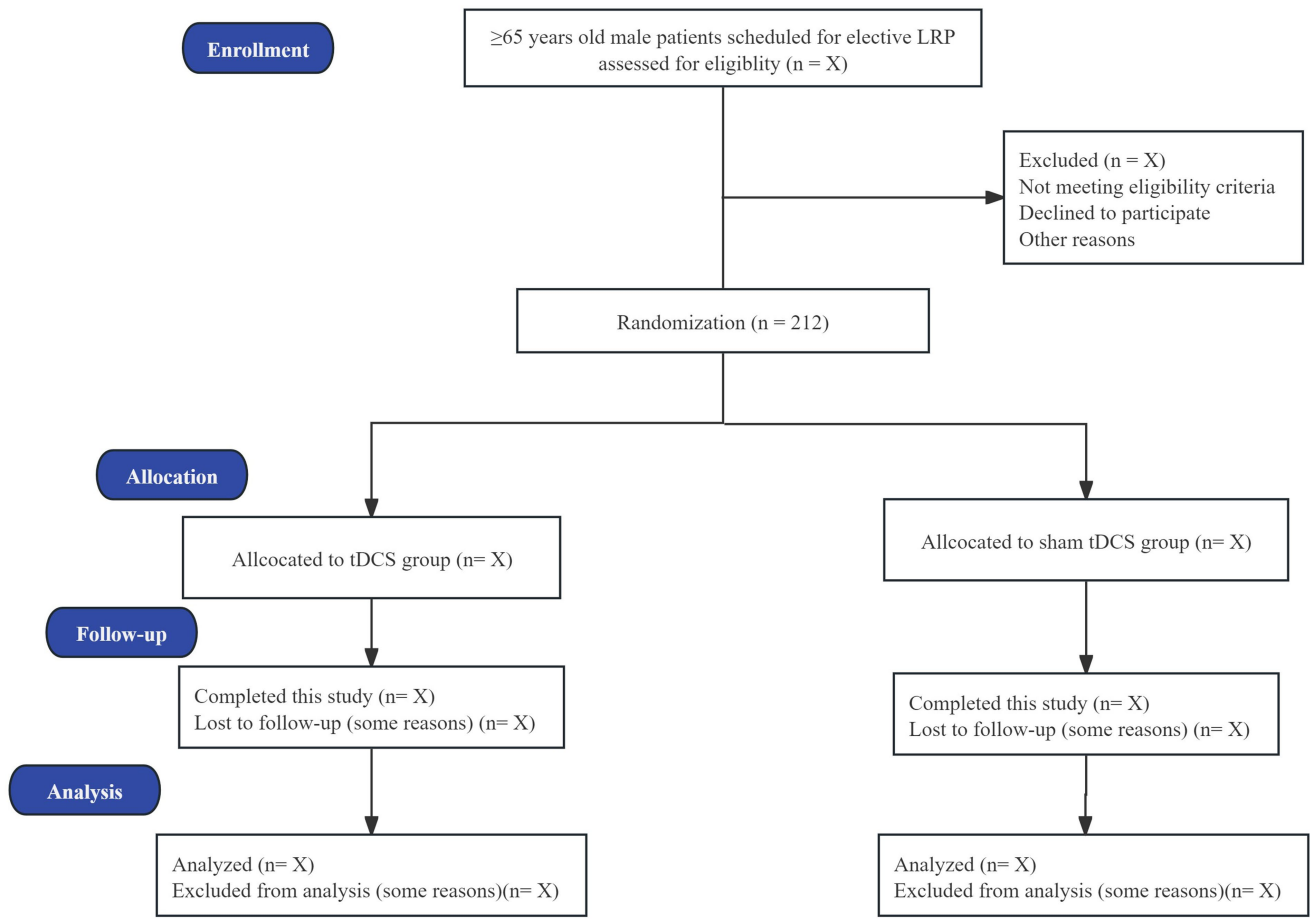
Flow chart of the study procedure. The diagram outlines the study design and participant timeline, encompassing enrollment, screening for eligibility, baseline assessment, and randomization. A total of 212 eligible elderly patients undergoing laparoscopic radical prostatectomy (LRP) will be randomly allocated in a 1:1 ratio to either the electroacupuncture (EA) group (*n* = 106) or the sham electroacupuncture (SEA) group (*n* = 106). The chart details the intervention phase, postoperative follow-up, assessment of primary and secondary outcomes (including incidence of POD, biomarkers, and adverse events), and final data collection. POD: Postoperative delirium.

This study is a two-center, randomized, sham-controlled trial with two parallel arms. The study protocol has been designed in accordance with the Standard Protocol Items: Recommendations for Interventional Trials (SPIRIT) 2013 statement (26). Eligible patients will be enrolled and randomly assigned in a 1:1 ratio to receive either EA or SEA. The trial is currently being conducted at the Affiliated Hospital of Jiaxing University and the Jiaxing Hospital of Traditional Chinese Medicine. Ethical approval was granted by the Ethics Committee of The Affiliated Hospital of Jiaxing University on July 30, 2024 (Approval No. 2024-KY-603) and by the Ethics Committee of the Jiaxing Hospital of Traditional Chinese Medicine on October 28, 2024 (Approval No. S1-2024-0096). The protocol is registered with the Chinese Clinical Trial Registry (Registration number: ChiCTR2500097337). A flowchart illustrating the study procedures is provided in Figure 1. Both groups will receive a standardized perioperative pain relief protocol to minimize confounding factors. Participant enrollment commenced in March 2025 and is anticipated to continue until July 2026. Written informed consent will be obtained from each participant prior to enrollment. The schedule of enrollment, interventions, and assessments is detailed in Table 1.

**Table 1.** Data collection methods and clinical assessment time points.

contents	Study period							
	T0	T1	T2	T3	T4	T5	T6	T7
ENROLLMENT								
Eligibility screening	X							
Informed consent	X							
Demographic characteristics		X						
Baseline measures		X	X					
Randomization	X							
INTERVENTION								
EA			X					
SEA			X					
ASSESSMENTS								
Incidence of POD				X	X	X	X	
Onset time of POD				X	X	X	X	
Duration of POD				X	X	X	X	
Severity of POD				X	X	X	X	
Subtypes of POD				X	X	X	X	
VAS scale				X	X	X	X	
Incidence of CRBD				X				
Opioid consumption						X	X	
Blood test indexes					X	X	X	
Perioperative anxiety score	X				X	X	X	
Safety outcomes	Throughout the study period until discharge							

T0:1 day of pre-surgery T1: 30 min before anesthesia T2: during operation T3:6h post-operation T4:1d post-operation T5: 2d post-operation T6:3d post-operation T7: hospital discharge

### Eligibility criteria

The inclusion and exclusion criteria for participants in this study are as follows:

Inclusion criteria

1) Male patients scheduled for elective LRP;
2) Participants were 65 years of age or older;
3) Participants with ASA classification I to III were included;
4) Participants are willing to provide written informed consent;

Exclusion criteria

1) Planned use of epidural or spinal anesthesia (i.e., non-general anesthesia);
2) Skin infection, open wounds, or severe dermatological conditions at the selected acupoints (ST36, GV20);
3) Pre-existing cognitive impairment, defined as a Mini-Mental State Examination (MMSE) score < 24;
4) Pre-existing diagnosis of dementia or other severe psychiatric disorders (e.g., schizophrenia) that may confound delirium assessment;
5) History of structural brain lesions with residual neurological deficits (e.g., traumatic brain injury, stroke within the past 3 months, intracranial tumors);
6) Presence of an implanted cardiac pacemaker or other implantable electronic devices (contraindication for EA);
7) Severe visual, auditory, or language impairments preventing effective communication and assessment;
8) Current history of alcohol abuse or drug dependence (to exclude withdrawal symptoms mimicking delirium);
9) Concurrent participation in another clinical trial.

### Randomization and blinding

Randomization will be performed by an independent statistician using SPSS software version 26.0, generating a random number sequence to assign participants in a 1:1 ratio to either the EA or SEA group. To ensure a balanced distribution between the two study sites, randomization will be stratified by center utilizing a block randomization method with varying block sizes of 4 or 6. The allocation sequence will be concealed in sequentially numbered, opaque, sealed envelopes, which will be kept in a secure location and opened by the acupuncturist only after the participant has provided informed consent and completed baseline assessments. Given the nature of the intervention, the acupuncturist cannot be blinded to group allocation; however, to minimize bias, this study employs a patient- and assessor-blinded design. Participants will remain blinded to their treatment assignment through the use of a sham procedure with identical device settings (lights and sound), while outcome assessors—including anesthesiologists responsible for intraoperative management and research registrars conducting postoperative delirium assessments—will be strictly blinded to group allocation. Additionally, the statistician performing the final analysis will handle data with coded identifiers to maintain blinding until the analysis is complete, with emergency unblinding permissible only if essential for clinical management of a severe adverse event.

To evaluate the integrity of the blinding procedures, a standardized blinding assessment questionnaire will be administered to all participants immediately following the completion of the intervention. Participants will be required to quantify the intensity of the stimulation sensation—encompassing specific De qi characteristics such as soreness, numbness, distension, or heaviness—and report their perception regarding the nature of the treatment received. The specific details this assessment are presented in Table 2.

**Table 2:** Questionnaire for the assessment of blinding effectiveness and acupuncture sensation.

Blind Method Assessment Questionnaire
Name:________ Random Number:_________ Date:___________ **Please answer the following questions according to the latest acupuncture experience.**
1. How’s your feel about needle sensation (sensation induced by acupuncture, like sourness, numbness, distending, heaviness, etc) Please ring a number between 0 (no needle sensation) and 10 (unbearable needle sensation) 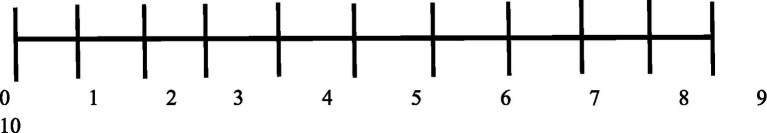 0 1 2 3 4 5 6 7 8 9 10
2. Do you have acupuncture experience? (Yes No)
3. Do you have electroacupuncture experience? (Yes No)
4. Are you sure you are receiving acupuncture treatment (Yes No)

### Intervention

A licensed acupuncturist with at least 3 years of clinical experience will perform the intervention. The protocol adheres to the Standards for Reporting Controlled Trials in Acupuncture (STRICTA) recommendations (27). Acupuncture will be performed 30 min prior to anesthesia induction. The acupoints Zusanli (ST36) and Baihui (GV20) were selected based on Traditional Chinese Medicine (TCM) theory and prior evidence suggesting their efficacy in regulating inflammatory responses and improving cerebral function, respectively.

**Figure 2:**
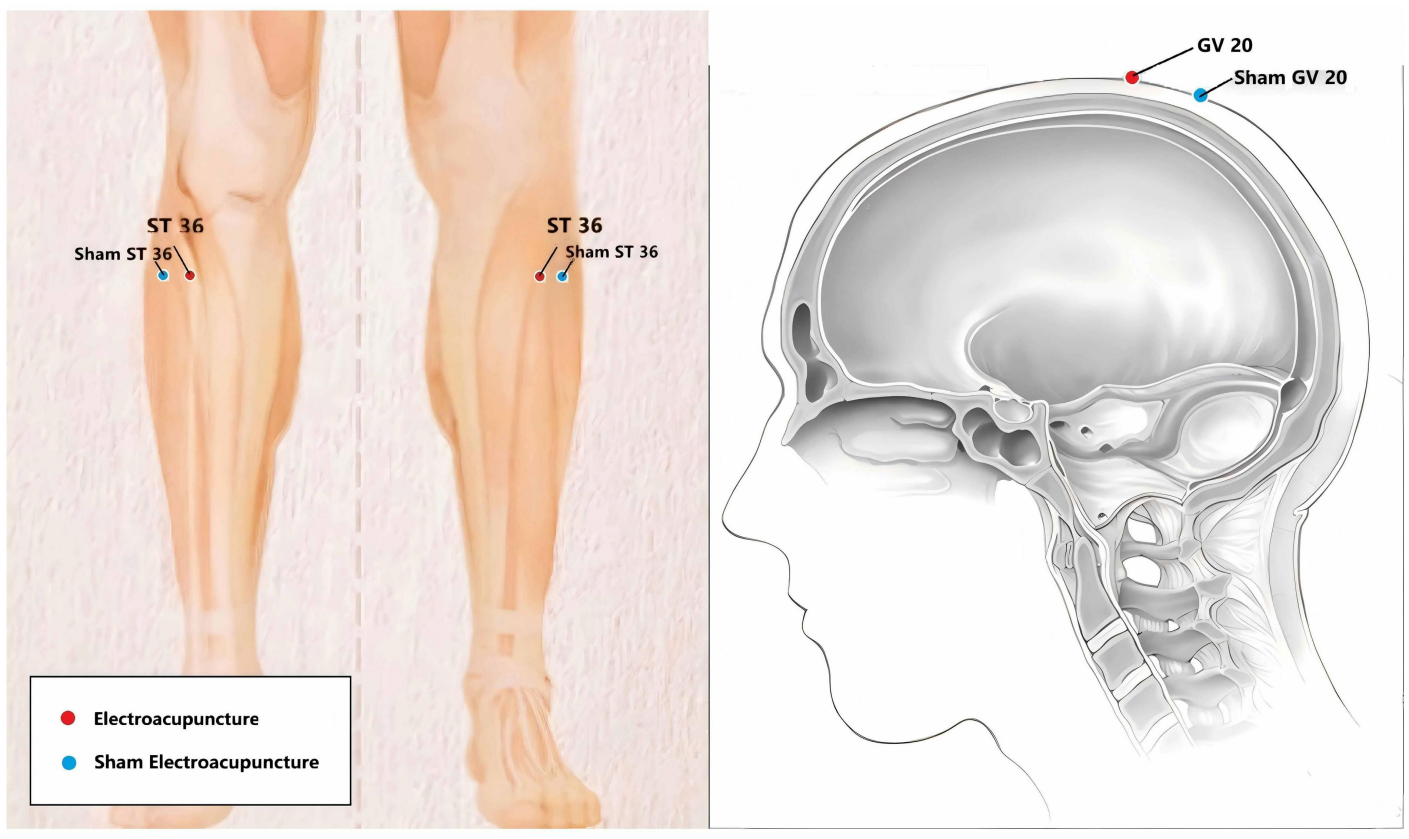
Anatomical location of acupoints for the Electroacupuncture (EA) and Sham Electroacupuncture (SEA) groups. The left panel illustrates the location of Zusanli (ST36) on the lower leg and its corresponding sham point. The right panel illustrates the location of Baihui (GV20) on the head and its corresponding sham point. Red dots indicate the true acupoints used in the EA group, while blue dots indicate the non-acupoints used in the SEA group (located approximately 20 mm lateral to the true acupoints).

Sterile, disposable stainless-steel needles (0.30 mm × 75 mm; Hwato Brand, Suzhou Medical Appliance Factory, China) will be inserted at the bilateral ST36 and GV20. Acupoint localization follows the WHO Standard (28), as detailed in Table 3 and Figure 2. The needles at ST36 will be inserted to a depth of 30–40 mm at a 30–45° angle inward and downward. The needle at GV20 will be inserted to a depth of 10–20 mm at a 15–30° angle posterior and downward. De qi sensation—characterized by soreness, numbness, distension, or heaviness—will be elicited by lifting, thrusting, and twirling the needles. Subsequently, the needles will be connected to an electro-stimulator (HANS-200A, Nanjing Jisheng Medical Co, Ltd) delivering a dilatational wave at 2 Hz. Stimulation will be maintained for 25–30 min. Any acupuncture-related adverse events, such as hematoma, severe pain, dizziness, or needle breakage, will be monitored and recorded.

**Table 3:** Location of ST36 and GV20.

Location of acupoints for the EA group	
Acupoint	Location
Zusanli (ST 36)	3 cun directly below Dubi (ST35), 1 finger-breadth lateral to the anterior border of the tibia
Bahui (GV 20)	Five cun directly above the midline of the frontal hairline, or at the midpoint of the line connecting the apex of both ears

1 cun (≈20 mm) is defined as the width of the interphalangeal joint of patient’s thumb.

Participants in the SEA group will receive a sham intervention. Needles will be inserted 20 mm lateral to ST36 and GV20 to a superficial depth of 3–4 mm without manipulation or elicitation of De qi. Although electrodes will be connected and the stimulator light activated to mimic the active group, no electrical current will be delivered. To ensure blinding integrity, patients in both groups will be informed that they may or may not feel the electrical stimulation.

### Anesthesia and perioperative management

Upon admission to the operating room, standard monitoring will be established, including electrocardiogram (ECG), heart rate (HR), pulse oxygen saturation (SpO2), and invasive blood pressure (IBP) via radial artery catheterization. Pre-oxygenation will be performed with 100% oxygen at 6 L/min. Anesthesia induction will be achieved via intravenous bolus injections of propofol (1–3 mg/kg), sufentanil (0.3–0.5 μg/kg), and rocuronium bromide (0.6–1 mg/kg). Following loss of consciousness and muscle relaxation, tracheal intubation will be performed by the attending anesthesiologist.

Anesthesia maintenance will be sustained with a continuous infusion of propofol (3–12 mg/kg/h) and remifentanil (0.1–0.3 μg/kg/min). Mechanical ventilation will be adjusted to maintain an end-tidal carbon dioxide (EtCO2) level of 35–45 mmHg. The administration of muscle relaxants (rocuronium) will cease approximately 30 min before the anticipated end of surgery. Additional sufentanil (0.1–0.2 μg/kg) and vasoactive medications will be administered as necessary to maintain hemodynamic stability. Additionally, at the end of the surgery, local anesthetic wound infiltration will be routinely performed by the surgeon at the trocar sites to optimize postoperative analgesia. After surgery, patients will be transferred to the post-anesthesia care unit (PACU). Postoperative pain management will be initiated using patient-controlled intravenous analgesia (PCIA) with sufentanil (concentration adjusted to deliver ~1 μg/kg total dose over the initial period, with a background infusion of 2 mL/h), supplemented by a bolus dose of 1.5 mL with a lockout interval of 15 min.

To minimize confounding factors associated with POD, several key perioperative variables will be strictly controlled. The depth of anesthesia will be monitored continuously using the Bispectral Index (BIS), targeting a range of 40–60. Core body temperature will be monitored via a nasopharyngeal probe, and forced-air warming systems will be utilized to maintain intraoperative normothermia (≥36.0 °C). Hemodynamics will be managed to keep the mean arterial pressure within 20% of the patient’s baseline value.

### Criteria for discontinuing or modifying allocated interventions

Based on the principles outlined in the Declaration of Helsinki (29), participants have the right to withdraw from the study at any stage and for any reason. Discontinuation of Intervention: The study intervention (EA or SEA) will be permanently discontinued under the following conditions:

1) Occurrence of severe adverse events (SAEs) related to the intervention (e.g., severe hematoma, infection, or needle breakage);
2) Vasovagal response (e.g., severe dizziness, syncope) during acupuncture;
3) Sudden hemodynamic instability (e.g., significant hypotension or arrhythmia) prior to anesthesia induction;
4) The participant requests to stop the intervention.

Modification of Intervention: If a participant experiences intolerance or discomfort (e.g., sharp pain) due to the electrical stimulation, the acupuncturist will first adjust the needle depth or reduce the current intensity to a tolerable level. If the discomfort persists despite adjustment, the stimulation will be ceased.

Data Collection for Discontinued Participants: For participants who discontinue the intervention but do not withdraw consent for data collection, follow-up assessments will continue as scheduled. Their data will be included in the final analysis based on the Intention-to-Treat (ITT) principle. For participants who withdraw consent for the entire study, no further data will be collected, but data obtained prior to withdrawal will be retained for safety analysis.

### Provisions for post-trial care

Following the completion of surgery, patients will be transferred to the PACU and subsequently to a general ward or ICU as clinically indicated. All participants will receive standard postoperative care provided by a multidisciplinary team comprising surgeons, anesthesiologists, and critical care specialists, regardless of their group allocation. In the specific event of harm or SAEs proven to be directly related to the study intervention (e.g., acupuncture-associated infection or injury), the research team is committed to providing the necessary medical treatment free of charge, in accordance with applicable laws and insurance policies. Throughout the postoperative period, investigators will conduct regular follow-up visits to closely monitor patient safety, detect potential complications, and provide timely clinical interventions. These visits also serve to address any participant concerns and ensure continuous medical support until discharge.

### Outcomes

#### Primary outcome

The primary outcome measure is the incidence of POD within the first three postoperative days. Assessments will be conducted by trained research registrars who are blinded to group allocation. The evaluation schedule commences 6 h after surgery and continues twice daily (between 08:00–10:00 and 16:00–18:00) from postoperative day 1 through day 3, or until discharge. At each assessment, the level of consciousness will first be screened using the Richmond Agitation-Sedation Scale (RASS). If the score is less than −3, assessment ceases, and the patient is documented as comatose. For patients with a RASS score of ≥ − 3, delirium will be evaluated using the 3-Minute Diagnostic Interview for CAM (3D-CAM) for ward patients, or the Confusion Assessment Method for the ICU (CAM-ICU) for those in the intensive care unit. The 3D-CAM employs CAM scale, comprising 22 items covering four diagnostic features: (1) acute fluctuations in mental status, (2) inattention, (3) disorganized thinking, and (4) altered level of consciousness. A positive diagnosis necessitates fulfilment of both (1) and (2) criteria, alongside at least one of (3) or (4) (30, 31). Any positive assessment during the observation period will be recorded as an incidence of POD.

#### Secondary outcome

1) Number of POD episodes: A positive 3D-CAM assessment will indicate the presence of a POD episode. Transition from POD to non-POD status will be established with two consecutive negative 3D-CAM assessments.
2) Onset time and duration of delirium: Onset time of delirium will be defined as the duration from the subject’s return to the surgical ward to the initial delirium diagnosis. Duration of delirium will include the period from the subject’s delirium diagnosis to the occurrence of two negative 3D-CAM assessments. The longest duration of delirium will be recorded for patients experiencing multiple episodes.
3) Delirium severity score: Patients with confirmed delirium will undergo assessment for delirium severity utilizing the Confusion Assessment Method-Severity (CAM-S) (32), with daily maximum scores documented. The CAM-S scale comprises four items, yielding a total score of 7 points. Severity grading includes absent (0), mild (1), or marked (2), with a total score of 0 indicating normalcy, 1 signifying mild delirium, 3–5 indicating moderate delirium, and 6–7 representing severe delirium.
4) Subtypes of delirium: Delirium subtypes—hyperactive, hypoactive, and mixed—will be determined based on clinical manifestations. RASS will be utilized to classify delirium subtypes, with hyperactive delirium characterized by consistently positive RASS scores (+1 to +4), hypoactive delirium denoted by consistently neutral or negative RASS scores (−3 to 0), and mixed delirium indicated when both positive and neutral/negative RASS scores are observed.
5) Pain scores: Postoperative pain at rest will be assessed using the Visual Analog Scale (VAS) during the scheduled afternoon assessment window (16:00–18:00) on postoperative days 1–3.
6) Opioid consumption: Opioid consumption, including PCIA and rescue analgesics, will be quantified on postoperative days 1 and 2. Opioid consumption will be converted to a “morphine equivalent dose” for analysis.
7) Blood test indexes: Venous blood samples (5 mL) will be collected at three time points: baseline (before acupuncture intervention/anesthesia induction), the end of surgery and postoperative day 1. Plasma levels of S100β and P-tau 217 proteins will be quantified via enzyme-linked immunosorbent assay (ELISA).
8) Incidence and severity of catheter-related bladder discomfort (CRBD): CRBD severity will be graded on a scale of 1 to 4, ranging from no discomfort to severe discomfort, with corresponding descriptions provided.
9) Perioperative anxiety score: Perioperative anxiety will be measured using the Hospital Anxiety and Depression Scale-Anxiety (HADS-A) subscale (33), assessing the incidence of perioperative anxiety from the day of surgery through 3 days postoperatively. The HADS-A consists of seven items and is a commonly used self-assessment scale for evaluating psychological distress in non-psychiatric patients, demonstrating good sensitivity and specificity. Patients with a HADS-A score of eight or above will be considered to have an anxiety disorder.
10) Adverse events: Safety endpoints include general postoperative complications (e.g., cardiac arrhythmias, hypotension, cardiogenic shock) and intervention-related adverse events specific to acupuncture (e.g., subcutaneous hematoma, needle site pain, dizziness/fainting, or needle breakage).

### Sample size

This study aims to investigate whether EA at specific acupoints can reduce the incidence of POD among elderly patients undergoing LRP. The sample size was calculated based on previous reports and our preliminary pilot study. According to earlier research, the incidence of POD in patients undergoing LRP is approximately 21%. In our preliminary pilot study involving 40 elderly patients undergoing LRP (*n* = 20 per group), the incidence of POD was 25% in the sham SEA group and 10% in the EA group. Furthermore, recent evidence in high-risk elderly populations has shown that electroacupuncture can significantly reduce the incidence of postoperative neurocognitive disorders, with reductions comparable to those observed in our pilot data (34). Considering these findings, it is hypothesized that the incidence of POD after treatment will decrease from a baseline of 20% to 8%. With a significance level set at 0.05 and a statistical power of 0.8, the required sample size to detect a significant difference between the two groups is 101 patients per group. Allowing for a potential 5% loss to follow-up, a total of 212 patients will be enrolled in this study.

### Additional consent provisions for collection and use of participant data and biological specimens

Informed consent will be obtained from all participants prior to enrollment. The investigators will provide a detailed explanation of the study procedures, including the EA intervention across the preoperative, intraoperative, and postoperative phases. The Informed Consent Form (ICF) explicitly outlines the scope of data collection and the specific procedures involved. Regarding biological specimens, a 5 mL venous blood sample will be collected at two time points: immediately before anesthesia induction and at the end of surgery. The ICF includes a specific provision regarding the collection, analysis, and storage of these specimens. Participants will be given the option to consent to—or decline—the preservation of their biological samples for potential future ancillary studies. Refusal to consent to future sample storage will not affect their participation in the main trial.

### Data collection and management

Data collection and management will be conducted in strict adherence to Good Clinical Practice (GCP) guidelines and applicable data protection regulations. Prior to study initiation, all researchers will undergo standardized training regarding the protocol, outcome assessment tools (specifically the 3D-CAM), and data entry procedures to ensure consistency. Data will be collected at preoperative, intraoperative, and postoperative time points and initially recorded on paper Case Report Forms (CRFs) derived from source documents. These CRFs will capture all required demographic, clinical (including specific perioperative variables such as BIS values, core temperature, intraoperative hemodynamics, estimated blood loss, and duration of pneumoperitoneum), and laboratory parameters, as well as adverse events. Subsequently, data will be transcribed into a secure, password-protected electronic database by designated investigators. To ensure data integrity and accuracy, a double-data entry strategy or independent verification process will be employed to identify and resolve any transcription errors. All participant information will be de-identified and assigned a unique study code; the key linking codes to personal identifiers will be stored separately in a locked cabinet accessible only to the principal investigator. Electronic files will be stored on a secure server with restricted access, ensuring that participant confidentiality is maintained throughout the study, analysis, and dissemination process.

### Plans for collection, laboratory evaluation and storage of biological specimens in this trial/future use

Venous blood samples (5 mL) will be collected by trained medical personnel at the designated baseline and postoperative time points. Immediately following collection, samples will be processed according to a standardized protocol, involving centrifugation to separate plasma, followed by aliquoting into coded, de-identified cryovials to prevent degradation from repeated freeze–thaw cycles. These specimens will be securely stored in a monitored ultra-low temperature freezer at −80 °C until the completion of participant enrollment. To minimize inter-assay variability, the quantification of plasma P-tau 217 and S100β levels will be performed using enzyme-linked immunosorbent assay (ELISA) techniques in batch analyses after all samples have been collected. Regarding future use, subject to the specific optional consent obtained from participants, any residual biological material will be preserved in a biobank for potential future ancillary studies investigating biomarkers of perioperative neurocognitive disorders.

### Statistical analysis

All analyses will be conducted according to the ITT principle, in which all randomized participants will be analyzed in the group to which they were originally assigned, regardless of protocol adherence or the treatment ultimately received. Baseline demographic and clinical characteristics will be summarized to assess the balance achieved between the two groups by randomization. Continuous variables will be presented as means with standard deviations (SDs) or medians with interquartile ranges (IQRs) based on the normality of distribution, while categorical variables will be presented as frequencies and percentages. Between-group comparisons for baseline data will be performed using the independent samples *t*-test, the Mann–Whitney U test, or the *χ*^2^ test (or Fisher’s exact test, as appropriate).

The primary outcome is a binary endpoint, defined as the incidence of POD within the first three postoperative days. The primary hypothesis will be tested using a generalized linear mixed model (GLMM) with a binary logistic link function to estimate the adjusted odds ratio (aOR) and 95% confidence interval (CI) for the EA group compared with the SEA group. The model will be adjusted for three pre-specified baseline covariates: age, ASA physical status, and baseline MMSE score. To account for the clustering effect inherent in the multicenter design, the study center will be included in the model as a random effect. To quantify the clinical effect size, a supplementary analysis will be conducted. We will use a modified Poisson regression model with a robust error variance to directly estimate the adjusted relative risk (aRR) after adjusting for the same covariates. The absolute risk reduction (ARR) and the number needed to treat (NNT), with their corresponding 95% CIs, will then be calculated from the aRR. Missing data on the primary outcome will be handled using multiple imputation by chained equations (MICE) under the missing-at-random assumption.

A comprehensive assessment of efficacy will be conducted by analyzing a series of secondary outcomes. For longitudinal continuous outcomes—such as the HADS-A scores measured at multiple time points—a linear mixed-effects model (LMM) will be used to account for within-subject correlation. For pre-to-post changes in biomarkers (P-tau 217 and S100β), an analysis of covariance (ANCOVA) model will be used, with the baseline value included as a continuous covariate to improve statistical precision. For all other secondary outcomes, continuous variables will be compared using the independent samples t-test or the Mann–Whitney U test; ordinal outcomes (such as CRBD severity) will be analyzed with the Mann–Whitney U test, and binary outcomes will be compared using the *χ*^2^ test or Fisher’s exact test. Safety outcomes, including the incidence of adverse events (e.g., acupuncture-related hematoma, dizziness) and general postoperative complications, will also be compared between the groups using the *χ*^2^ test or Fisher’s exact test. Additionally, to assess the success of blinding, the agreement between participants guessed and actual treatment allocation will be quantified using Cohen’s Kappa coefficient.

Two exploratory analyses will be conducted. First, to identify potential predictors of POD, we will perform separate univariable logistic regression analyses for key intraoperative variables (e.g., duration of anesthesia, estimated blood loss), reporting unadjusted odds ratios and 95% CIs. Second, we will investigate the association between these variables and the treatment effect to generate hypotheses for future research.

We will perform a series of subgroup analyses of the primary outcome to explore the potential heterogeneity of the treatment effect (i.e., effect modification) across patient characteristics. These analyses will be based on age, ASA physical status, and baseline MMSE score. These analyses will be performed by including an interaction term between the treatment assignment and the subgrouping variable in the primary GLMM model. We will primarily focus on the *p* value for the interaction to assess for statistically significant effect modification and present the aORs and 95% CIs for each subgroup in a forest plot. To test the robustness of the primary findings under different analytical assumptions, we will conduct a series of sensitivity analyses. First, to assess the influence of protocol deviations, we will repeat the primary analysis on the Per-Protocol (PP) population. Second, to evaluate the overall impact of covariate adjustment, we will fit a univariable model containing only the treatment assignment and compare its effect estimate (crude odds ratio) with that of our primary multivariable model (aOR). Third, to assess the influence of the statistical modeling of the center effect, we will repeat the analysis treating the study center as a fixed effect rather than a random effect. Finally, to address potential bias from data missing not at random, a “worst-case” scenario analysis will be performed, assuming all missing outcomes in the EA group are events and all in the SEA group are non-events.

## Discussion

This double-center, patient- and assessor-blinded, randomized controlled trial aims to evaluate the efficacy of EA preconditioning at acupoints ST36 and GV20 in reducing the incidence of POD among elderly patients undergoing LRP. We hypothesize that the EA intervention will not only reduce the clinical incidence of POD but also reduce the postoperative elevation of plasma P-tau 217 and S100β levels. To our knowledge, this study will be the first to investigate the potential of EA in this specific surgical population and to explore its association with these neuroprotective biomarkers. If our hypothesis is confirmed, this trial could provide clinical evidence for a non-pharmacological strategy to improve postoperative cognitive outcomes in elderly urological patients.

Previous research on POD has predominantly focused on major orthopaedic and cardiac surgeries, with less attention paid to urological populations (35, 36). Given the advanced age typical of prostate cancer patients, this population is at heightened risk for POD, which is strongly correlated with adverse outcomes, including increased mortality (37, 38). Therefore, the prevention and management of POD in this group is clinically significance. However, current data regarding POD following LRP are limited to small-sample studies, which often present methodological variations. To address this, the present trial uses a randomized controlled design. We identified potential confounding variables from the literature—such as age, ASA classification, education level, comorbidities, and lifestyle factors—and employed stratified randomization and blinding protocols to ensure a balanced distribution, thereby minimizing selection and assessment bias.

Recent studies suggest the efficacy of EA in improving cognitive dysfunction (39–41). Regarding the acupoints selected for this study, previous research has confirmed the effects of acupuncture at ST36 and GV20 on animal models of cognitive dysfunction (42, 43). According to TCM meridian theory, GV20 is pivotal for ‘awakening the upper’ to regulate the mind, and when combined with ST36 (which functions to ‘regulate the middle’ and strengthen the foundation), they are used to treat cognitive deficits (44). Recent research using P301S Tau transgenic mice demonstrated that EA stimulation at GV20 disrupts the interaction between tau pathology and neuroinflammation by suppressing the TNF-α/NF-κB/NLRP3 signaling axis, thereby protecting cognitive function (45). Ni et al. showed that in an AD animal model, EA stimulation at ST36 alleviated cognitive impairment and β-amyloid pathology by inhibiting the activation of the NLRP3 inflammasome (46). These mechanisms are relevant to POD, which involves neuroinflammation and neurodegeneration. This study will therefore investigate whether EA preconditioning can reduce the postoperative increase in P-tau and S100β. We hypothesize that by suppressing this inflammatory response, the intervention may alleviate postoperative cognitive dysfunction and reduce the incidence of POD.

This study has several limitations. First, the primary endpoint is restricted to the first three postoperative days; consequently, the long-term effects of EA on neurocognitive recovery (e.g., at 3, 6, or 12 months) will not be assessed in this trial. Second, the anticipated effect size used for sample size calculation was derived from a small-scale pilot study. Although this pilot data is supported by similar findings in other high-risk surgical populations, the true effect size in the current double-center trial might be smaller than our optimistic estimate. Consequently, this study may be underpowered if the actual treatment effect is less pronounced than projected. Finally, as this is a two-center trial conducted exclusively in China, the generalizability of the findings to different ethnic populations or healthcare systems may be limited. Future research should therefore focus on long-term neurocognitive follow-up, incorporate a wider array of biomarkers to fully elucidate the underlying pathways, and include multinational trials to confirm these benefits across diverse populations.

## Glossary

EA: Electroacupuncture
LRP: Qlaparoscopic radical prostatectomy
POD: Postoperative delirium
P-tau: Phosphorylated tau
CRBD: Catheter-related bladder discomfort
ASA: American Society of Anaesthesiologists
MMSE: Mini-Mental State Examination
CAM: Confusion assessment method
STRICTA: Standards for Reporting Controlled Trials in Acupuncture
ECG: Electrocardiogram
HR: Heart rate
IBP: Invasive blood pressure
PACU: Post-anaesthesia awakening recovery unit
VAS: Visual analogue scale
DSM-V: The Diagnostic and Statistical Manual of Mental Disorders criteria
RASS: Richmond Agitation Sedation Scale
CRF: Case record form
ITT: Intention-to-treat
PP: Per-protocol
OR: Odds ratio
CI: Confidence interval
BDNF: Brain-derived neurotrophic factor
AD: Alzheimer’s disease
Aβ: Amyloid beta
